# 1477. Do Federal Risk Assessment Criteria Adequately Classify Patients for Surgical Site Infections Following Colon Surgery?

**DOI:** 10.1093/ofid/ofad500.1313

**Published:** 2023-11-27

**Authors:** Yasna Yusuf, Briana Episcopia, Mary Fornek, Marie Abdallah, John Quale

**Affiliations:** New York city health and hospitals/kings County, Brooklyn, New York; NYC H+H/ Kings County, Garden City, New York; New York City Health and Hospital, Manhattan, New York; New York City Health and Hospital/Kings County, Brooklyn, New York; New York City Health and Hospital/Kings County, Brooklyn, New York

## Abstract

**Background:**

Infections related to colon surgery are associated with adverse outcomes. Colon SSIs are reportable to National Healthcare Safety Network (NSHN) and Centers for Medicare and Medicaid Services (CMS). Rates of SSIs are factored into federal reimbursement programs; financial penalty may exist for “low-performing” hospitals. CMS employs a risk assessment tool to calculate the number of expected colon SSIs for each center.

**Methods:**

The NYC H+H consist of hospitals that serve patients of low socioeconomic status. Five of the eleven are designated as level 1 trauma centers. Data regarding patients that underwent colon surgery from 2015-2022, including those that developed SSIs, were obtained from NHSN. Propensity score matching was done to match patients with colon SSIs to those without SSIs. Covariates included the CMS risk factors: gender, diabetes, age, BMI, ASA score, surgical closure type.

**Results:**

A total of 5217 patients underwent colon surgery from 2015-2022. There were 276 patients with a colon SSI. A propensity matched cohort (n=276), using the CMS risk assessment variables, was matched with the 276 patients with SSIs (Table 1).

Other features involving the colon surgery were examined for the two cohorts (Table 2). Compared to the matched controls, there was a disproportionate distribution of wound class groups among the SSI cohort. Significantly more patients had dirty wounds in the SSI group. Surgery was more often emergent, and had a significantly longer duration in the SSI cohort. Of the patients with SSIs, more were trauma cases, and a significantly greater percentage had surgery performed in a trauma center compared to the other hospitals.

**Figure:**
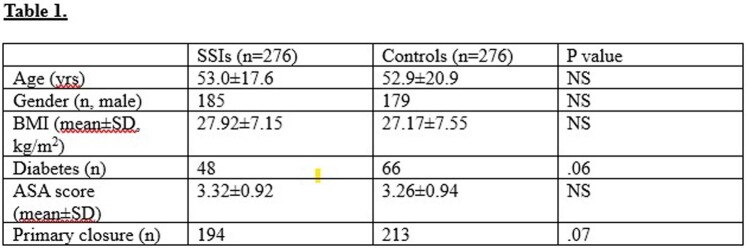

**Figure:**
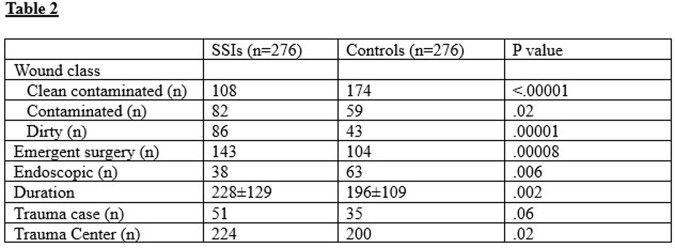

**Conclusion:**

Colon SSI preventive measures are not applicable for emergent and trauma cases. The current risk assessment model used by CMS does not fully adjust for the complexity of various colon surgeries. As such, hospital performance as judged by federal SSI assessments does not correlate with the quality of care provided. Financial penalties imposed by CMS may be devastating, particularly for safety net hospitals serving patients primarily of low socioeconomic status. Until appropriate risk assessment models are developed, the use of colon surgery SSIs as a quality measure should be re-evaluated.

**Disclosures:**

**All Authors**: No reported disclosures

